# Yellow urticaria secondary to acalabrutinib-induced liver injury

**DOI:** 10.1093/omcr/omad142

**Published:** 2024-01-27

**Authors:** Simran Kalsi, Sabrina Bennett, Deborah L Cook, Alyssa Fischer

**Affiliations:** Department of Medicine, Division of Dermatology, Larner College of Medicine at the University of Vermont, 89 Beaumont Avenue, Burlington, VT, USA; Department of Medicine, Division of Dermatology, University of Vermont Medical Center, 111 Colchester Avenue, Burlington VT, USA; Department of Pathology and Laboratory Medicine, Division of Dermatopathology, University of Vermont Medical Center, 111 Colchester Avenue, Burlington VT, USA; Department of Medicine, Division of Dermatology, University of Vermont Medical Center, 111 Colchester Avenue, Burlington VT, USA

## CLINICAL IMAGE

**Figure 1:**
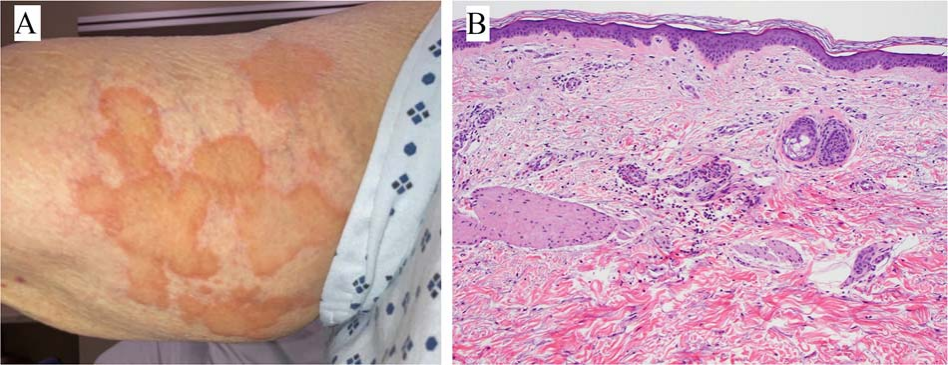
(**A**) Right arm with annular and serpiginous, edematous, yellowish plaques with erythematous borders. (**B**) Sparse perivascular and interstitial polymorphous infiltrate composed of neutrophils, eosinophils, and lymphocytes (H&E, 200×).

A 79-year-old man with CLL, polymyalgia rheumatica, and hyperlipidemia was admitted with hyperbilirubinemia (total bilirubin 2.8 mg/dl), and transaminitis (AST 736 U/l, ALT 1023 U/l) one month after switching from ibrutinib to acalabrutinib. Presenting symptoms included a 1-week history of fatigue, malaise, abdominal pain, nausea, vomiting, diarrhea, and a new rash. Physical examination showed multiple yellow urticarial plaques on the back, right arm (Fig. 1A), and left thigh. Dermatology was consulted and performed a shave biopsy of the rash. Histopathology revealed perivascular and interstitial dermatitis consistent with urticaria (Fig. 1B). Viral hepatitis and congestive hepatopathy were excluded, suggesting acalabrutinib-induced liver injury with yellow urticaria as a presenting sign. Acalabrutinib was discontinued and treatment began with cetirizine 20 mg twice daily and diphenhydramine nightly as needed. At one month follow-up, the rash had resolved and transaminase levels normalized. Yellow urticaria is a rare variant of urticaria caused by transient vascular permeability and deposition of bilirubin in the skin forming wheals [1, 2]. Hyperbilirubinemia due to hepatitis, cirrhosis, acute liver failure, and metastatic liver disease may result in yellow urticaria [2]. Diagnosis of yellow urticaria is clinical and treatment is symptomatic. In this case, acalabrutinib was likely the catalyst for developing liver injury and yellow urticaria which both resolved upon discontinuation of the drug. Asymptomatic or mild elevations in serum aminotransferase levels are a known adverse effect of acalabrutinib, but severe elevation to above 5 times ULN only occurs in 2%–3% of patients and rarely results in drug discontinuation [3]. The patient continues to follow with hematology and has deferred resuming treatment for their CLL.
